# Role of platelet gene polymorphisms in ischemic pediatric stroke subtypes: a case-control study

**DOI:** 10.3325/cmj.2020.61.18

**Published:** 2020-02

**Authors:** Andrea Čeri, Jasna Leniček Krleža, Désirée Coen Herak, Marija Miloš, Marina Pavić, Nina Barišić, Vlasta Đuranović, Renata Zadro

**Affiliations:** 1 Department of Medical Biochemistry and Hematology, University of Zagreb Faculty of Pharmacy and Biochemistry, Zagreb, Croatia; 2 Department of Laboratory Diagnostics, Children's Hospital Zagreb, Zagreb, Croatia; 3 Department of Laboratory Diagnostics, University Hospital Centre Zagreb, Zagreb, Croatia; 4 Department of Laboratory Diagnostics in Traumatology and Orthopedics, Clinical Institute of Chemistry, University Hospital Centre Sestre Milosrdnice, Zagreb, Croatia; 5 Division of Neuropediatrics, Department of Pediatrics, University Hospital Centre Zagreb, Zagreb, Croatia; 6 Department of Neuropediatrics, Children's Hospital Zagreb, Zagreb, Croatia

## Abstract

**Aim:**

To assess the role of human platelet antigens (HPA), P-selectin gene (*SELP*) polymorphisms, and HPA and *SELP* haplotypes with factor V (*FV*) R506Q in ischemic pediatric stroke (IPS) subtypes: cerebral sinovenous thrombosis (CSVT), perinatal (PAIS), and childhood (CAIS) arterial ischemic stroke.

**Methods:**

This case-control study enrolled 150 children with confirmed IPS and 150 age- and sex-matched controls. *FV* R506Q and HPA-1 were genotyped with CVD StripAssay®, HPA-2 and HPA-3 with real-time polymerase chain reaction, *SELP* S290N, V599L, and T715P with high resolution melting analysis, and *SELP* N562D with sequence-specific polymerase chain reaction.

**Results:**

HPA-1b allele (odds ratio [OR] 2.75, 95% confidence interval [CI] 1.02-7.42, *P* = 0.048) and HPA-1a2a3b (OR 5.46, 95% CI 1.51-19.76, *P* = 0.011), HPA-1b2a3a (OR 7.00, 95% CI 1.25-39.13, *P* = 0.028), and HPA-1b2b3a (OR 11.39, 95% CI 1.39-92.95, *P* = 0.024) haplotypes increased the risk for CSVT. HPA-3b allele was significantly associated with 2-fold lower risk for PAIS (OR 0.49, 95% CI 0.26-0.89, *P* = 0.020) and CAIS (OR 0.47, 95% CI 0.26-0.86, *P* = 0.014) and non-significantly associated with increased risk for CSVT (OR 6.43, 95% CI 0.83-50.00, *P* = 0.022). HPA-1a2b3a haplotype was significantly associated with CAIS (OR 6.76, 95% CI 2.13-21.44, *P* = 0.001). The inclusion of *FV* R506Q in *SELP* haplotype analysis increased the risk for PAIS 4-fold in QNDVT carriers (OR 8.14, 95% CI 0.93-71.33, *P* = 0.060) compared with NDVT haplotype (OR 2.45, 95% CI 0.98-6.18, *P* = 0.058), but the result was not significant.

**Conclusion:**

Individual HPAs, and particularly HPA haplotypes, are involved in IPS subtypes pathogenesis. A possible risk-inducing synergistic effect of *SELP* haplotypes with *FV* R506Q is restricted to PAIS only.

Ischemic pediatric stroke (IPS) is a relatively rare heterogeneous multifactorial disorder caused by arterial (ie, arterial ischemic stroke, AIS) or venous occlusion (ie, cerebral sinovenous thrombosis, CSVT). According to the time of stroke onset, AIS is classified as perinatal (PAIS) and childhood AIS (CAIS) (1,2). IPS subtypes differ in incidence rates, etiology, presentation symptoms, and treatment strategies (2,3), and their predisposing disorders are still incompletely understood and characterized (4-6). Risk factors for IPS include various inherited and acquired prothrombotic disorders (2,4,5). However, the role of different genetic risk factors in the etiology of IPS subtypes has been studied in a limited number of publications, and studies including multiple genetic factors and haplotype analysis are extremely rare.

The most frequently investigated genetic risk factor is the polymorphism in factor V gene (*FV*) that causes amino acid change R506Q (FV Leiden, NM_000130.4:c.1601G>A, rs6025) and consequently activated protein C resistance and susceptibility to thrombosis (7). *FV* R506Q has been regularly associated with IPS, although in CSVT the association is weaker in children than in adults (4,8-10).

Platelets have a significant role in maintaining normal hemostasis. Changes in the structure of platelet membrane proteins can change platelet function and predisposition to thrombophilia. The effect of variations in platelet glycoprotein receptor genes and the P-selectin adhesion molecule on their role in IPS has not been established yet (11).

Human platelet antigens (HPA) are genetically defined polymorphisms expressed on platelet membrane glycoproteins. In three out of six biallelic systems, ie, HPA-1 (NM_000212.2:c.176T>C, rs5918) on glycoprotein IIIa, HPA-2 (NM_000173.5:c.482C>T, rs6065) on glycoprotein Ibα, and HPA-3 (NM_000419.3:c.2621T>G, rs5911) on glycoprotein IIb, a base-pair substitution leads to amino acid change in a platelet surface membrane glycoprotein. These biallelic systems modulate platelet receptor density, altering platelet function and thrombus formation (12-14). The role of HPAs in ischemic stroke has been recognized, but poorly investigated in adults (15-18) and particularly in children (9,19-21).

P-selectin mediates the interaction of activated endothelial cells or platelets with leukocytes (22,23). Multiple polymorphisms in P-selectin gene (*SELP*) have been described, but only five of them cause amino acid substitution that may influence its function: V168M (NM_003005.3:c.625G>A, rs6125), S290N (NM_003005.3:c.992G>A, rs6131), N562D (NM_003005.3:c.1807G>A, rs6127), V599L (NM_003005.3:c.1918G>T, rs6133), and T715P (NM_003005.3:c.2266A>C, rs6136) (24). *SELP* polymorphisms appear to be associated with several stages of thrombosis and associated diseases, including venous thromboembolism and atherothrombotic disease (25-27), cardiovascular disease, and myocardial infarction in adults (24,28-31). Although the relationship of different *SELP* polymorphisms to ischemic stroke in adults has been described (32-37), there are no reports regarding their role in IPS.

Since IPS subtypes have different pathophysiologic backgrounds, it is justified to investigate the relative relationship between thrombophilia polymorphisms and stroke subtypes. Therefore, the aim of this study was to assess the role of eight individual polymorphisms (*FV* R506Q, HPA-1, HPA-2, HPA-3, *SELP* S290N, N562D, V599L, and T715P) and their haplotypes (HPA-1/-2/-3, *SELP* S290N/N562D/V599L/T715P, and *FV* R506Q /*SELP* S290N/N562D/V599L/T715P) in IPS subtypes: PAIS, CAIS, and CSVT.

## Participants and methods

### Participants

This case-control study enrolled 150 children aged up to 18 years with a confirmed diagnosis of PAIS, CAIS, or CSVT and 150 age- and sex-matched controls from the same geographical region with no history of thromboembolic or neurological events and with normal C reactive protein levels. Controls were recruited among children undergoing minor surgery such as tonsillectomy and children with respiratory diseases at routine follow-up visits. All children were admitted to the University Hospital Centre Zagreb or Children’s Hospital Zagreb, Zagreb, Croatia, from 1999 to 2018. The recruitment dynamics was five patients per year until 2004, with increasing tendency of seven to nine patients per year afterwards for AIS; one case of CSVT per year was recruited from 2008 to 2010 and three cases per year from 2013 to 2017.

The diagnosis was established after an extensive analysis of patients’ medical history and physical and neurological examination; it was based on the presence of clinical symptoms and signs and confirmed by at least one brain imaging technique. Isolated computed tomography scans were used in selected cases only (N = 9) during the first recruitment years. Magnetic resonance imaging was performed in 141 patients; in 72 to confirm computed tomography scan findings and in 69 patients, in the later phase of research, as the only technique used. AIS was diagnosed based on the presence of neurological deficit of acute onset, seizures, or other signs of neonatal encephalopathy, and confirmed by neuroradiographic findings of parenchymal infarcts in cerebral arteries accordant with clinical manifestations. PAIS and CAIS were differentiated according to the definitions by Lynch (2).

CSVT was diagnosed after the neuroradiographic confirmation of a thrombus or flow interruption within cerebral veins or dural sinuses, together with clinical presentations of headache, seizure, lethargy, and focal or generalized neurologic deficit (38). Patients were included after a definite CSVT diagnosis by a neuroradiologist based on computed tomography as the first imaging exam for excluding a tumor, subdural hematoma, or abscess followed by magnetic resonance imaging combined with magnetic resonance angiography and venography as currently the best method for the confirmation of CSVT.

Written informed consent was obtained from all participants’ parents and additionally from all children older than 12 years. The study was conducted in accordance with the tenets of the Declaration of Helsinki and approved by the Ethics Committee for Experimentation of the University of Zagreb Faculty of Pharmacy and Biochemistry (251-62-03-14-95), Ethics Committee of the University Hospital Centre Zagreb (02/21/JG), and Ethics Committee of the Children’s Hospital Zagreb (01-26/18-14).

### Molecular analysis

Genomic DNA was isolated from peripheral blood leukocytes and used for molecular analysis (Table 1). *FV* R506Q and HPA-1 were genotyped with CVD StripAssay® T and CVD StripAssay® A (ViennaLab Diagnostics, Vienna, Austria), respectively. Both tests were performed according to manufacturer’s instructions: each DNA sample was amplified in two parallel multiplex polymerase chain reactions (PCR) using biotin-labeled primers. Amplification products were selectively hybridized to a test strip containing allele-specific oligonucleotide probes immobilized as an array of parallel lines. Bound PCR fragments were detected using streptavidin-alkaline phosphatase conjugate and color substrates. HPA-2 and HPA-3 were genotyped using previously described real-time PCR method based on TaqMan® technology on 7500 Real-Time PCR System (Applied Biosystems, Foster City, CA, USA) (39). Three positive controls representing different genotypes for each polymorphism were included in each run as a quality control step (40). Polymorphisms *SELP* S290N, V599L, and T715P were genotyped using PCR with specific primers followed by high resolution melting analysis on the LightCycler® 480 Real-Time PCR System (Roche Diagnostics, Mannheim, Germany) and results were analyzed using LightCycler® 480 Software (version 1.5; Roche Diagnostics) (41). Control samples of each genotype previously confirmed by sequencing were used as positive controls (24). *SELP* N562D was genotyped using the previously described PCR with sequence specific primers on the Applied Biosystems GeneAmp 2720 Thermal Cycler (24).

**Table 1:** Genotyping methods for eight individual polymorphisms*

Polymorphism	Method	Primer and minor groove binding probe sequences	Reference
*FV* R506Q	multiplex PCR using biotin labeled primers, hybridization to ASO probes	n.a.	–
HPA-1	multiplex PCR using biotin labeled primers, hybridization to ASO probes	n.a.	–
HPA-2	real-time PCR method based on TaqMan® technology	F: 5′-GAGCTCTACCTGAAAGGCAATGA-3′ R: 5′-TGTTGTTAGCCAGACTGAGCTTCT-3′ Pa: 5′-VIC-CTCCTGACGCCCACAC-NFQ-3′ Pb: 5′-FAM-CTCCTGATGCCCACAC-NFQ-3′	(39)
HPA-3	real-time PCR method based on TaqMan® technology	F: 5′-GCCTGACCACTCCTTTGCC-3′ R: 5′-TGCGATCCCGCTTGTGA-3′ Pa: 5′-VIC-CTGCCCATCCCCA-NFQ-3′ Pb: 5′-FAM-CTGCCCAGCCCCA-NFQ-3′	(39)
*SELP* S290N	PCR with specific primers followed by high resolution melting analysis	F: 5′-CCTTGGTTATTCTCTCCAGCTGTGC-3′ R: 5′-AGCCGGGCTGGCACTCAAAT-3′	(41)
*SELP* N562D	PCR with sequence specific primers	FN: 5′-CTCCACCTGYCATTTCTCTTGTA-3′ FD: 5′-CTCCACCTGYCATTTCTCTTGTG-3′ R: 5′-AAGTAGAACTGTCTTAGCAAGTAC-3′	(24)
*SELP* V599L	PCR with specific primers followed by high resolution melting analysis	F: 5′-TTGCAGGAGCCTCCCTTGTTATGAA-3′ R: 5′-GGTTCCCTGCCCAGGAGTGGT-3′	(41)
*SELP* T715P	PCR with specific primers followed by high resolution melting analysis	F: 5′-ATGAACTGCTCCAACCTCTG-3′ R: 5′-CCCACATGAAAATTGTACCTT-3′	(41)

*ASO – allele-specific oligonucleotide; n.a. – not applicable; *FV* – factor V gene; HPA – human platelet antigens; *SELP*- P – selectin gene; PCR – polymerase chain reaction; NFQ – nonfluorescent quencher.

### Statistical analysis

Normality of distribution was tested with the Shapiro-Wilk test (MedCalc software package version 9.3.2.0, Frank Schoonjans, the Netherlands). Continuous variables are expressed as medians and ranges. Descriptive analysis, Hardy-Weinberg equilibrium testing, and association analysis were performed with SNPStats (Catalan Institute of Oncology, Barcelona, Spain) (42,43), a web-based tool for the analysis of association studies that analyzes single SNPs by multiple inheritance models and multiple SNPs (haplotype analysis) based on logistic regression. The obtained genotyping results of *FV* R506Q, *SELP* polymorphisms, and HPAs, *SELP* S290N/N562D/V599L/T715P, *FV* R506Q/*SELP* S290N/N562D/V599L/T715P, and HPA-1/-2/-3 haplotypes are expressed as frequencies. Hardy-Weinberg equilibrium was tested for each individual polymorphism in patients and controls. The most common combined genotypes were used as reference haplotypes in the analysis of haplotype association with disease. Associations of each individual polymorphism and haplotype with the disease risk were expressed as odds ratios (OR) with corresponding 95% confidence intervals (CI) by using a dominant model (a homozygous or heterozygous variant in comparison with the homozygous wild-type). A *P* value of <0.050 was considered significant.

## Results

All IPS subtypes were more prevalent in boys (Table 2). Genotype distributions of all investigated individual polymorphisms, both in cases and controls, were in Hardy-Weinberg equilibrium (results not shown), except HPA-3 polymorphism in children with PAIS (*P* = 0.035).

**Table 2:** Characteristics of patients with ischemic stroke and the control group*

Group	Number of participants	Male to female ratio	Age at diagnosis in years, median (range)	Age at testing in years, median (range)
IPS	150	1.54	1.9 (0.0-18.0)	4.6 (0.0-18.0)
AIS	132	1.59	1.9 (0.0-18.0)	5.9 (0.0-18.0)
PAIS	66	1.36	0.3 (0.0-12.0)	2.7 (0.0-18.0)
CAIS	66	1.87	6.8 (0.4-18.0)	7.9 (0.4-18.0)
CSVT	18	1.25	1.4 (0.0-12.8)	2.0 (0.0-15.4)
Control group	150	1.54	–	7.0 (0.0-18.0)

*Dash – not applicable; IPS – ischemic pediatric stroke; AIS – arterial ischemic stroke; PAIS – perinatal arterial ischemic stroke; CAIS – childhood arterial ischemic stroke; CSVT – cerebral sinovenous thrombosis.

Among the examined individual polymorphisms, IPS was associated with only three polymorphisms: *FV* R506Q, HPA-1, and HPA-3 (Table 3). PAIS was significantly associated with *FV R506Q*; *FV R506Q* carriers had 4-fold increased risk for PAIS. Carriers of at least one HPA-1b allele had a 2.75-fold increased risk for CSVT, while carriers of at least one HPA-3b allele had an approximately 2-fold lower risk for IPS and AIS, including both PAIS and CAIS. Carriers of HPA-3b allele (OR 6.43, 95% CI 0.83-50.00, *P* = 0.022; data not shown) had an increased risk for CSVT, but the result was not significant. However, additive model revealed a 2.23-fold increased risk for CSVT (95% CI 1.04- 4.80, *P* = 0.034).

**Table 3:** Individual polymorphisms in patients with ischemic pediatric stroke and its subtypes, and in the control group*

Polymorphism name	Patient group	Genotype	Genotype distribution in patient group, frequency	Genotype distribution in control group, frequency	OR (95% CI)	P
*FV* R506Q	PAIS	GG	0.879	0.967	4.00 (1.26-12.74)	0.017
		GA	0.121	0.033		
HPA-1	CSVT	aa	0.500	0.733	2.75 (1.02-7.42)	0.048
		ab	0.500	0.247		
		bb	0.000	0.020		
HPA-3	IPS	aa	0.411	0.287	0.58 (0.36-0.93)	0.025
		ab	0.411	0.507		
		bb	0.178	0.207		
	AIS	aa	0.457	0.287	0.48 (0.29-0.78)	0.003
		ab	0.388	0.507		
		bb	0.155	0.207		
	PAIS	aa	0.453	0.287	0.49 (0.26-0.89)	0.020
		ab	0.344	0.507		
		bb	0.203	0.207		
	CAIS	aa	0.461	0.287	0.47 (0.26-0.86)	0.014
		ab	0.431	0.507		
		bb	0.108	0.207		

*OR – odds ratio; CI – confidence intervals; IPS – ischemic pediatric stroke; *FV* – factor V gene; AIS – arterial ischemic stroke; PAIS – perinatal arterial ischemic stroke; HPA – human platelet antigen; CSVT – cerebral sinovenous thrombosis; CAIS – childhood arterial ischemic stroke.

Carriers of HPA-1a2b3a had a 4-fold increased risk for IPS and AIS, and 7-fold for CAIS. Interestingly, three different HPA-1/-2/-3 haplotypes showed a significant association with CSVT, resulting in five- to 11-fold increased risk: HPA-1a2a3b, HPA-1b2a3a, and HPA-1b2b3a. Haplotype HPA-1a2b3b was found in children with CSVT (0.054) and control group (0.050) only, but the result was not significant (OR 2.70, 95% CI 0.32-22.46, *P* = 0.360; data not shown) (Table 4).

**Table 4:** Human platelet antigen HPA-1/-2/-3 haplotype frequencies in patients with ischemic pediatric stroke and its subtypes, and the control group

Group	HPA-1/-2/-3 haplotype	Haplotype frequency	OR (95% CI)	P
IPS	HPA-1a2a3a	0.429	1.00 (Ref.)	Ref.
AIS		0.446	1.00 (Ref.)	Ref.
PAIS		0.485	1.00 (Ref.)	Ref.
CAIS		0.429	1.00 (Ref.)	Ref.
CSVT		0.138	1.00 (Ref.)	Ref.
Control group		0.433	–	–
IPS	HPA-1a2a3b	0.301	0.89 (0.59-1.34)	0.570
AIS		0.285	0.79 (0.51-1.21)	0.270
PAIS		0.323	0.86 (0.52-1.41)	0.540
CAIS		0.250	0.70 (0.39-1.24)	0.220
CSVT		0.542	5.46 (1.51-19.76)	0.011
Control group		0.351	–	–
IPS	HPA-1b2a3a	0.078	1.45 (0.64-3.27)	0.370
AIS		0.079	1.32 (0.59-2.94)	0.500
PAIS		0.071	1.10 (0.43-2.79)	0.850
CAIS		0.086	1.54 (0.60-3.94)	0.370
CSVT		0.137	7.00 (1.25-39.13)	0.028
Control group		0.056	–	–
IPS	HPA-1b2a3b	0.073	1.06 (0.52-2.18)	0.870
AIS		0.064	0.91 (0.43-1.92)	0.810
PAIS		0.052	0.74 (0.28-1.95)	0.550
CAIS		0.074	1.10 (0.44-2.75)	0.840
CSVT		0.044	3.17 (0.39-25.93)	0.280
Control group		0.060	–	–
IPS	HPA-1a2b3a	0.094	3.97 (1.29-12.17)	0.016
AIS		0.104	4.46 (1.49-13.37)	0.008
PAIS		0.056	2.15 (0.58-8.02)	0.250
CAIS		0.147	6.76 (2.13-21.44)	0.001
CSVT		0.016	4.19 (0.11-154.50)	0.440
Control group		0.023	–	–
IPS	HPA-1b2b3a	0.016	0.57 (0.11-2.99)	0.510
AIS		0.013	0.37 (0.05-2.58)	0.320
PAIS		0.014	0.45 (0.05-3.73)	0.460
CAIS		0.015	0.42 (0.04-3.99)	0.450
CSVT		0.068	11.39 (1.39-92.95)	0.024
Control group		0.027	–	–

*Dash – not applicable; Ref. – reference haplotype; HPA – human platelet antigen; OR – odds ratio; CI – confidence intervals; IPS – ischemic pediatric stroke; AIS – arterial ischemic stroke; PAIS – perinatal arterial ischemic stroke; CAIS – childhood arterial ischemic stroke; CSVT – cerebral sinovenous thrombosis.

Six *SELP* S290N/N562D/V599L/T715P and of *FV* R506Q /*SELP* S290N/N562D/V599L/T715P haplotypes were identified in all study groups. Although haplotype NDVT was more frequent in children with AIS and PAIS and least frequent in CAIS and AIS compared with control group, the result was not significant. Three rare haplotypes, NNLT, RNNLT, and QNDVT, were identified in AIS and controls only (results not shown). Haplotype QNDVT was more common in patients with PAIS than in control group (0.040 vs 0.002), but the result was not significant (OR 8.14, 95% CI 0.93-71.33, *P* = 0.060) (Table 5).

**Table 5:** *SELP* S290N/N562D/V599L/T715P and *FV* R506Q/*SELP* S290N/N562D/V599L/T715P haplotype frequencies in patients with ischemic pediatric stroke and its subtypes, and the control group*

Group	*SELP* S290N/N562D/V599L/T715P haplotype	Haplotype frequency	OR (95% CI)	P	*FV* R506Q/*SELP* S290N/N562D/V599L/T715P haplotype	Haplotype frequency	OR (95% CI)	P
IPS	SDVT	0.396	1.00 (Ref.)	Ref.	RSDVT	0.405	1.00 (Ref.)	Ref.
AIS		0.387	1.00 (Ref.)	Ref.		0.397	1.00 (Ref.)	Ref.
PAIS		0.413	1.00 (Ref.)	Ref.		0.419	1.00 (Ref.)	Ref.
CAIS		0.381	1.00 (Ref.)	Ref.		0.386	1.00 (Ref.)	Ref.
CSVT		0.445	1.00 (Ref.)	Ref.		0.448	1.00 (Ref.)	Ref.
Control group		0.405	–	–		0.393	–	–
IPS	SNVT	0.267	1.18 (0.74-1.87)	0.490	RSNVT	0.259	1.18 (0.73-1.89)	0.500
AIS		0.271	1.19 (0.74-1.93)	0.460		0.267	1.20 (0.74-1.95)	0.450
PAIS		0.266	1.18 (0.65-2.12)	0.590		0.259	1.19 (0.66-2.16)	0.570
CAIS		0.271	1.19 (0.66-2.14)	0.560		0.270	1.16 (0.64-2.09)	0.620
CSVT		0.249	0.96 (0.36-2.53)	0.930		0.219	0.82 (0.30-2.28)	0.700
Control group		0.240	–	–		0.240	–	–
IPS	NDVT	0.100	1.86 (0.83-4.17)	0.130	RNDVT	0.065	1.47 (0.61-3.52)	0.390
AIS		0.115	2.10 (0.93-4.75)	0.076		0.074	1.64 (0.67-4.01)	0.280
PAIS		0.108	2.45 (0.98-6.18)	0.058		0.061	1.89 (0.67-5.38)	0.230
CAIS		0.102	1.84 (0.68-5.00)	0.230		0.079	1.48 (0.51-4.30)	0.470
CSVT		0.041	0.48 (0.05-4.22)	0.510		0.040	0.47 (0.05-4.16)	0.500
Control group		0.070	–	–		0.067	–	–
IPS	NNVT	0.073	0.57 (0.28-1.17)	0.130	RNNVT	0.068	0.53 (0.25-1.13)	0.100
AIS		0.058	0.48 (0.22-1.05)	0.068		0.051	0.44 (0.19-1.00)	0.051
PAIS		0.063	0.48 (0.18-1.26)	0.140		0.049	0.40 (0.14-1.18)	0.098
CAIS		0.057	0.45 (0.15-1.36)	0.160		0.057	0.47 (0.16-1.39)	0.170
CSVT		0.154	1.45 (0.44-4.77)	0.540		0.155	1.44 (0.43-4.78)	0.550
Control group		0.108	–	–		0.109	–	–
IPS	SNVP	0.077	1.07 (0.57-2.01)	0.840	RSNVP	0.069	1.08 (0.58-2.04)	0.800
AIS		0.086	1.17 (0.61-2.24)	0.630		0.076	1.20 (0.63-2.29)	0.580
PAIS		0.032	0.70 (0.28-1.76)	0.450		0.028	0.76 (0.30-1.90)	0.550
CAIS		0.121	1.66 (0.80-3.48)	0.180		0.121	1.62 (0.77-3.40)	0.200
CSVT		0.008	0.37 (0.05-2.88)	0.340		0.007	0.37 (0.05-2.85)	0.340
Control group		0.077	–	–		0.077	–	–
IPS	SNLT	0.074	1.13 (0.52-2.44)	0.750	RSNLT	0.075	1.04 (0.48-2.21)	0.930
AIS		0.077	1.22 (0.56-2.68)	0.620		0.078	1.12 (0.52-2.45)	0.770
PAIS		0.087	1.44 (0.57-3.69)	0.440		0.088	1.32 (0.52-3.37)	0.560
CAIS		0.068	1.05 (0.40-2.74)	0.930		0.064	0.89 (0.33-2.42)	0.820
CSVT		0.056	0.81 (0.14-4.55)	0.810		0.056	0.79 (0.14-4.44)	0.790
Control group		0.066	–	–		0.066	–	–

*Dash – not applicable; Ref. – reference haplotype; *FV* – factor V gene; *SELP* – P-selectin gene; OR – odds ratio; CI – confidence intervals; IPS – ischemic pediatric stroke; AIS – arterial ischemic stroke; PAIS – perinatal arterial ischemic stroke; CAIS – childhood arterial ischemic stroke; CSVT – cerebral sinovenous thrombosis.

## Discussion

This study demonstrated that various HPA genotypes and haplotypes were associated with IPS subtypes in a sample from Croatian child population and corroborated the hypothesis that different IPS subtypes did not share the same genetic risk factors.

The present study revealed an almost 3-fold increased risk for pediatric CSVT in carriers of at least one HPA-1b allele. This makes it the first study to our knowledge that found a positive association of HPA-1 and pediatric CSVT. The only study that investigated HPA-1 in pediatric CSVT to date reported more frequent HPA-1b allele-containing genotypes in CSVT than in both AIS and control group, but the results were not significant (20).

Consistent with previous findings on a moderate protective effect of HPA-3b allele for AIS and PAIS in Croatian population (9), the present study reported a 2-fold lower risk for IPS and CAIS, with the *post-hoc* calculated power of 0.51 for PAIS, 0.54 for CAIS, 0.59 for IPS, and 0.82 for AIS with a significance level of 0.050. On the contrary to this, we identified an unexpectedly high harmful effect of HPA-3b allele in CSVT, as opposed to its protective effect in AIS, but these findings did not reach significance. Although the sample size for CSVT is small, the polymorphism frequency is high. *Post-hoc* power analysis revealed the power of 0.44, meaning that additional 25 participants are needed to obtain the optimal power of 0.80.

HPA-1 and HPA-3 are both present on the most abundant glycoprotein IIb/IIIa complex, which, by binding fibrinogen, is essential for platelet aggregation and thrombus formation. Recently, Ichord has reported that major risk factors for CSVT are acute head and neck infections as well as acute systemic illness (44), a conclusion similar to that reached in our study. As acute illness is linked to higher fibrinogen level, enhanced platelet-fibrinogen interactions are possible, leading to the formation of clots that are more stable and resistant to lysis.

A further analysis of HPA-1/-2/-3 haplotypes demonstrated for the first time that particular haplotypes were positively associated with both AIS and CSVT. Haplotype HPA-1a2b3a conferred an almost 7-fold increased risk for CAIS, but not for PAIS or CSVT, with a consequent increased risk for AIS and IPS. Moreover, compared with the effect of HPA-1 alone, two HPA-1b allele containing haplotypes, HPA-1b2a3a and HPA-1b2b3a, conferred a three- to 4-fold increased risk for CSVT, respectively, whereas HPA-1a2a3b haplotype conferred a slightly lower risk.

Studies on HPAs in IPS are rare, provide contradictory results, and include only HPA-1 in differently defined pediatric populations (19-21). Literature search revealed an association of four specific HPA-1/-2/-3/-4 haplotypes with adult ischemic stroke and HPA-1b/2b/3a haplotype with coronary arterial disease, but the results are not comparable to our study due to differences in studied populations and HPAs included in haplotype analysis (17,45).

Concordant with the majority of studies performed in adults (32-37), the present study found no association between individual *SELP* polymorphisms and any IPS subtype. The *post-hoc* power analysis revealed very low power for all individual *SELP* polymorphisms in IPS. Although no significant association between *SELP* S290N/N562D/V599L/T715P haplotypes and adult ischemic stroke was identified in Caucasian population (34), the present study revealed an increased presence of the NDVT haplotype in children with AIS and PAIS, but not in children with CAIS and CSVT, pointing to its possible role in the etiology of PAIS only. The effect of NDVT haplotype may be explained by a previously reported association of *SELP* S290N/N562D/T715P haplotype NDT with increased soluble P-selectin plasma concentrations and the fact that *SELP* polymorphisms S290N and N562D are located within the SELP region important for the binding of P-selectins on leukocytes (26,46). As NNVT haplotype tends to decrease the risk for AIS, while NDVT haplotype tends to increase the risk for PAIS and AIS, it seems that N562D polymorphism is crucial for conferring the susceptibility to AIS.

The present study confirmed the association of *FV* R506Q with PAIS, which was previously established in a smaller study (8,46). Since the presence of multiple risk factors can have a synergistic effect (47), additional *SELP* haplotype analysis also included *FV* R506Q, an established risk factor for IPS, which is located in the close proximity. Although the statistical significance was still not achieved, the inclusion of *FV* R506Q increased the risk for PAIS 4-fold in QNDVT carriers, as compared with NDVT haplotype alone, indicating a possible synergistic effect of *SELP* haplotype and *FV* R506Q.

The strength of this study is the inclusion of all IPS subtypes, including CSVT, enabling differentiation of their specific etiologies based on simultaneous identification of both harmful and protective genotype combinations. Moreover, the inclusion of haplotype analysis proved to be superior to the testing of single polymorphisms. To our knowledge, this is the only study to date investigating a possible association of *SELP* polymorphisms and HPA-1/-2/-3, *SELP* S290N/N562D/V599L/T715P and *FV* R506Q/*SELP* S290N/N562D/V599L/T715P haplotypes with IPS.

The study limitations include the relatively small sample of children with IPS, as association studies usually require large cohorts to minimize possible statistical biases in conclusions and to strengthen the study power. Certainly, the results obtained for only 18 CSVT cases should be taken with caution, but nevertheless, they present the preliminary evidence of the positive association of individual HPAs and the enhanced risk-inducing effect of the HPA-1/-2/-3 haplotypes for CSVT. Considering the aforementioned and the variable geographical and ethnical distribution of HPA genotypes (48), we cannot claim that the associations presented in this study can be applied to different populations or that the effect is limited to the Croatian population only, warranting further research of platelet gene polymorphisms in IPS subtypes.

To evaluate genotype-phenotype associations, the present study used the candidate gene approach rather than genome-wide approach because of the lower cost and higher statistical power, especially if genes likely to play a role in the examined disease are formerly known, which is important for small-scale studies. Genome-wide association studies can reveal new genes or gene combinations even when their function was not previously known, but they usually require extensive funding and have low power due to the number of independent tests performed (49).

Our findings indicate that different IPS subtypes are characterized by specific sets of inherited thrombophilia risk factors and that there is a variable role of polymorphisms in the etiology of IPS subtypes. In the era of personalized medicine, it is crucial to better understand the clinical value and physiological implications of different genetic entities if we want to treat patients properly and reduce morbidity and mortality. We believe that future trials with sample sizes increased through international collaborations and extensive haplotype analysis would achieve a greater power to confirm the role of all the examined haplotypes in the etiology of IPS subtypes.
